# Screening for Intimate Partner Violence in an Oncology Population

**Published:** 2013-11-01

**Authors:** Lydia T. Madsen

**Affiliations:** Ms. Madsen is an advanced practice nurse in the department of Genitourinary Medical Oncology, The Univeristy of Texas MD Anderson Cencer Center, Houston, Texas. Dr. McFarlane is a profesor and Parry Nursing Chair in Health Promotion in the College of Nursing, Texas Woman’s University

Intimate partner violence (IPV) is most broadly defined as behavior that is abusive and perpetrated by someone who is in a current or previous relationship with the victim (Nelson, Bougatsos, & Blazina, 2012); see Table 1. Intimate partner violence may occur on a continuum ranging from isolated incidents described as situational couple violence to intimate terrorism encompassing multiple aspects of psychological and physical abuse (Johnson, 2008). Although few studies have focused solely on the oncology population, a 2006 study by Modesitt and colleagues states that a staggering 75% of women under treatment for breast, ovarian, endometrial, or ovarian cancer report having experienced some form of intimate partner abuse during adulthood (Modesitt et al., 2006). Results from a 2002 National Violence Against Women Survey confirmed this high rate of incidence (Canady, Naus, & Babcock, 2010).

**Table 1:**
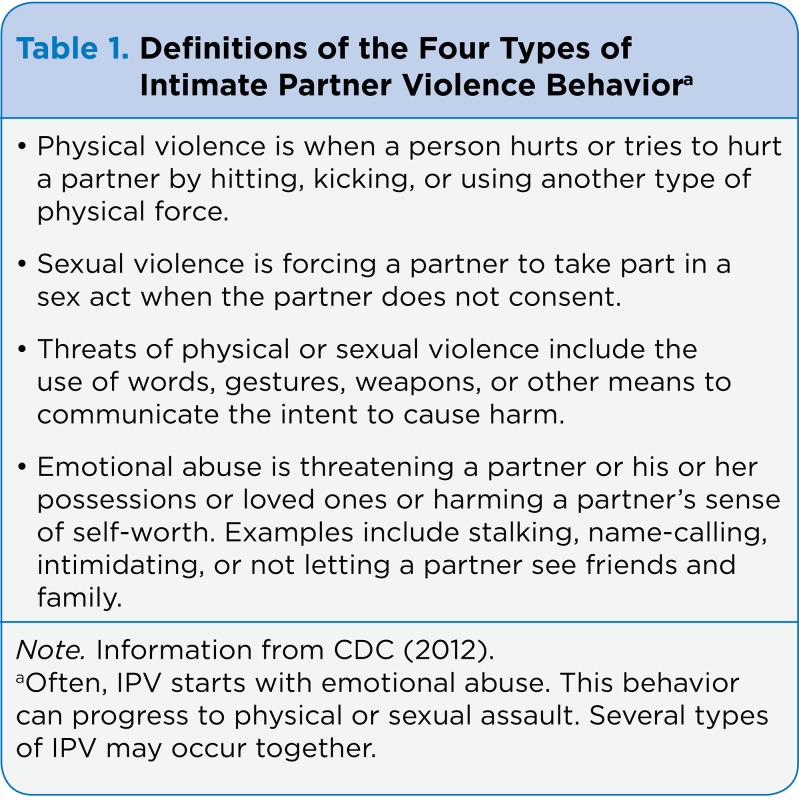
Table 1. Definitions of the Four Types of Intimate Partner Violence Behavior

## Screening Recommendations

In January 2013, the US Preventive Services Task Force (USPSTF) issued a recommendation for health-care providers to begin routine screening of women patients for IPV (USPSTF, 2013). The USPSTF’s recommendation aligns with the 2011 recommendation by the Institute of Medicine that all women of childbearing age should be routinely screened by their health-care provider for IPV (Kottenstette & Stulburg, 2013). These recommendations are based on current evidence that screening and intervention in the health-care setting reduce both the incidence of IPV and the related health outcomes. Intimate partner violence and oncology intersect because IPV occurrence is a public health concern; the reported incidence of IPV ranges from 22% to 39% over a woman’s lifetime. This high rate of incidence greatly increases the likelihood that oncology patients may experience IPV in addition to their cancer diagnosis (Cesario, in press).

Although current recommendations are for screening women of childbearing age, older women also have the potential to be vulnerable to IPV (Sawin & Parker, 2011). In addition to the likelihood that older women may remain in an abusive relationship because of financial dependence, older women are also at increased risk to sustain injury if physical abuse and neglect occur (Sawin & Parker, 2011).

## Frequently Used Tools

No single IPV screening tool is routinely used in practice or has, to date, well-established psychometric properties (Rabin, Jennings, Campbell, & Bair-Merritt, 2009). However, the most frequently used screening tools in this review included the Women’s Experience with Battering (WEB) Scale and the Psychological Maltreatment of Women Inventory (Short-Form); see Appendices A and B on pages 459 and 460. Both tools provide basic screening questions for patients in the clinical setting (Tolman, 1989). These screening tools provide the additional benefit of sensitivity in screening for emotional IPV (Sawin & Parker, 2011).

Owen-Smith and colleagues (2008) identified advanced practitioners as the health-care providers most able to integrate IPV screening into their practice. Advanced practitioners routinely assess oncology patients in the outpatient setting and facilitate referrals, which may include psychosocial support. Advanced practitioners also routinely spend significant time with the patient assessment, allowing for an opportunity to develop rapport and encourage domestic violence disclosure (Owen-Smith et al., 2008). Survivors of IPV recommend repeated screenings, as this routine may facilitate future disclosure of abuse (Owen-Smith et al., 2008). In fact, a literature review with case examples by Schmidt, Woods, and Stewart (2006) noted that in each of the case studies presented, neither the oncologist nor the nurses identified the abuse based on injury or suspicious behavior; patients had been referred to psychiatry for "evaluation of mood."

## Routine Approach

Screening for IPV may be uncomfortable for health-care providers, so using a routine approach for all female patients may be most efficacious; providing a statement disclosing that the policy of the health-care provider is to screen all female patients diminishes the burden of trying to implement a targeted screening. When health-care providers routinely assess for IPV as part of the standard psychosocial assessment, they should (a) be aware of their state laws for reporting a positive screening, (b) remain nonjudgmental and supportive when IPV screening is positive and provide resource information, and (c) facilitate referral to the appropriate social services when indicated (Mick, 2006). Resources for the advanced practitioner and the patient can be found in Table 2.

**Table 2:**
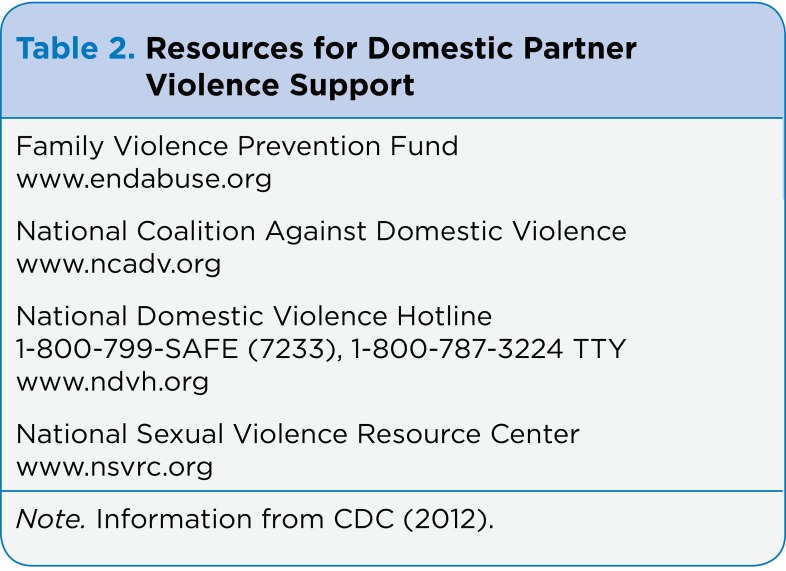
Table 2. Resources for Domestic Partner Violence Support

**Table 3:**
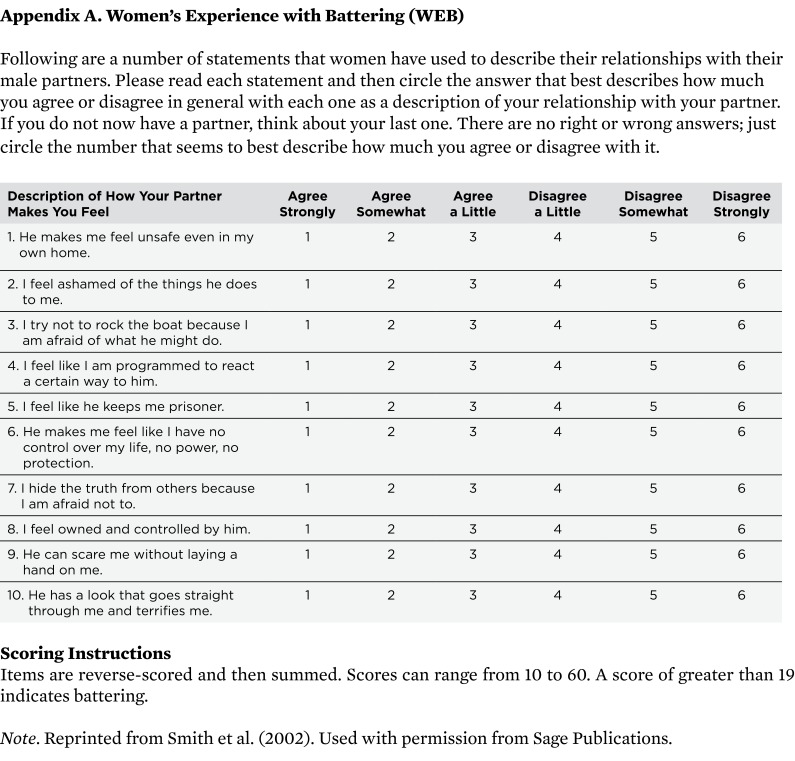
Appendix A. Women's Experience with Battering (WEB)

**Table 4:**
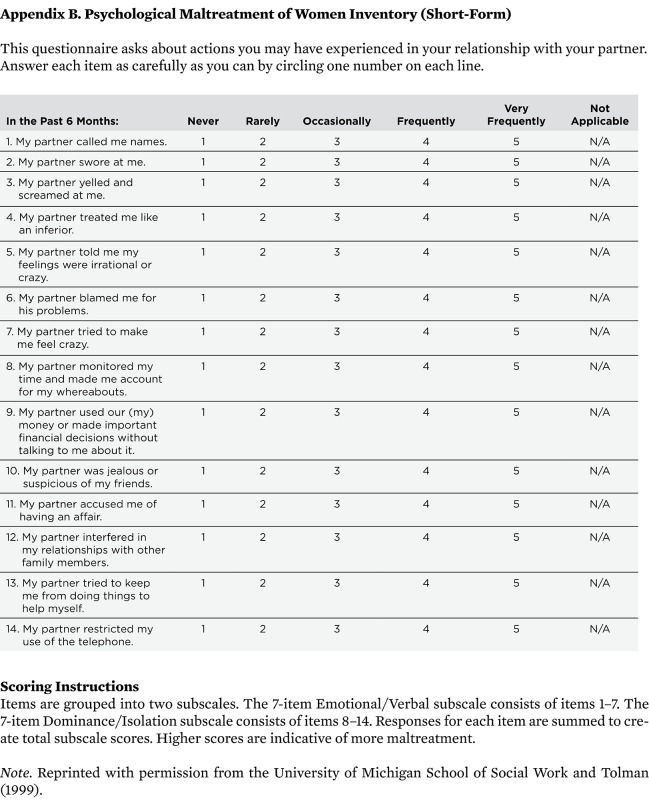
Appendix B. Psychological Maltreatment of Women Inventory (Short-Form)

## References

[1] Canady Brittany E, Naus Mary J, Babcock Julia C (2010). Physical and psychological abuse in breast cancer survivors and cancer-free women.. *Journal of psychosocial oncology*.

[2] (2012). Understanding intimate partner violence. *Centers for Disease Control and Prevention.*.

[3] (2013). Measuring intimate partner violence victimization and perpetration: A compendium of assessment tools. *Centers for Disease Control and Prevention*.

[4] Cesario Sandra K, McFarlane Judith, Nava Angeles, Gilroy Heidi, Maddoux John (2014). Linking cancer and intimate partner violence: the importance of screening women in the oncology setting.. *Clinical journal of oncology nursing*.

[5] Johnson M. P.  (2008). *A typology of domestic violence: Intimate terrorism, violent resistance and situational couple violence*.

[6] Kottenstette Jennifer Bello, Stulberg Debra (2013). PURLs: time to routinely screen for intimate partner violence?. *The Journal of family practice*.

[7] Mick JoAnn (2006). Identifying signs and symptoms of intimate partner violence in an oncology setting.. *Clinical journal of oncology nursing*.

[8] Modesitt Susan C, Gambrell Alisa C, Cottrill Hope M, Hays Lon R, Walker Robert, Shelton Brent J, Jordan Carol E, Ferguson James E (2006). Adverse impact of a history of violence for women with breast, cervical, endometrial, or ovarian cancer.. *Obstetrics and gynecology*.

[9] Nelson H. D., Bougatsos C., Blazina I.  (2012). Screening women for intimate partner violence: A systematic review to update the U.S. Preventive Services Task Force Recommendation.. *Annals of Internal Medicine*.

[10] Owen-Smith Ashli, Hathaway Jeanne, Roche Maria, Gioiella Marie Elena, Whall-Strojwas Denise, Silverman Jay (2008). Screening for domestic violence in an oncology clinic: barriers and potential solutions.. *Oncology nursing forum*.

[11] Rabin  R. F., Jennings J. M., Campbell J. C., Bair-Merritt M. H. (2009). Intimate partner violence screening tools. *American Journal of Preventive Medicine*.

[12] Sawin Erika Metzler, Laughon Kathryn, Parker Barbara J, Steeves Richard H (2009). Breast cancer in the context of intimate partner violence: a qualitative study.. *Oncology nursing forum*.

[13] Sawin E. M., Parker B. (2011). If looks would kill then I would be dead: Intimate partner abuse and breast cancer in older women. *Journal of Gerontological Nursing*.

[14] Schmidt N. K., Woods  T. E., Stewart J. A. (2006). Domestic violence against women with cancer: Examples and review of the literature.. *Journal of Supportive Oncology*.

[15] Smith P H, Earp J A, DeVellis R (1995). Measuring battering: development of the Women's Experience with Battering (WEB) Scale.. *Women's health (Hillsdale, N.J.)*.

[16] Smith P. H., Thornton G. E., DeVellis R., Earp J., Coker A. L. (2002). A population-based study of the prevalence and distinctiveness of battering, physical assault, and sexual assault in intimate relationships. *Violence Against Women*.

[17] Tolman R M (1989). The development of a measure of psychological maltreatment of women by their male partners.. *Violence and victims*.

[18] Tolman R M (1999). The validation of the Psychological Maltreatment of Women Inventory.. *Violence and victims*.

[19] (2013). Screening for intimate partner violence and abuse of elderly and vulnerable adults. *US Preventive Services Task Force.*.

